# A Noise Level Prediction Method Based on Electro-Mechanical Frequency Response Function for Capacitors

**DOI:** 10.1371/journal.pone.0081651

**Published:** 2013-12-09

**Authors:** Lingyu Zhu, Shengchang Ji, Qi Shen, Yuan Liu, Jinyu Li, Hao Liu

**Affiliations:** 1 State Key Laboratory of Electrical Insulation and Power Equipment, Xi'an Jiaotong University, Xi'an, Shaanxi, China; 2 Shaoxing Electric Power Bureau, Shaoxing, Zhejiang, China; University of Adelaide, Australia

## Abstract

The capacitors in high-voltage direct-current (HVDC) converter stations radiate a lot of audible noise which can reach higher than 100 dB. The existing noise level prediction methods are not satisfying enough. In this paper, a new noise level prediction method is proposed based on a frequency response function considering both electrical and mechanical characteristics of capacitors. The electro-mechanical frequency response function (EMFRF) is defined as the frequency domain quotient of the vibration response and the squared capacitor voltage, and it is obtained from impulse current experiment. Under given excitations, the vibration response of the capacitor tank is the product of EMFRF and the square of the given capacitor voltage in frequency domain, and the radiated audible noise is calculated by structure acoustic coupling formulas. The noise level under the same excitations is also measured in laboratory, and the results are compared with the prediction. The comparison proves that the noise prediction method is effective.

## Introduction

With the rapid development of high-voltage direct-current (HVDC) transmission, the number of capacitors in HVDC converter stations and the harmonic currents flowing through the capacitors increase dramatically, leading to a great increase of audible noise coming out from the capacitors [1]. The noise may cause serious impact on the life of people around, such as disturbing their peace and endangering their health. Control of the noise has been an important task for researchers and engineers. If the noise level is predicted accurately before a converter station is constructed, corresponding measures against the noise can be taken in advance. Therefore, study of noise level prediction methods is of research value and engineering significance.

Much research has been devoted to studying the characteristics and prediction methods of the capacitor noise. Cox and Guan calculated the vibration responses of capacitor surface under distorted capacitor current based on the transfer functions obtained from impact hammer tests, but the noise level of the capacitors was measured instead of calculated [2]. Smede et al, in their experiment, established a 1:4 scaled acoustic model of capacitor stack to study the characteristics of noise [3]. Obviously, this method is uneconomical, time-consuming and tedious. In our previous paper [4], a formula was presented to calculate the capacitor noise level based on vibration velocity of capacitor surface. However, the calculation of the vibration was not concerned. We have also presented a method for calculating the noise level of capacitors based on modal analysis and impact hammer experiment [5]. This method is currently the most adopted approach by the major manufacturers in China in predicting the noise level when designing converter stations. However, in the impact hammer experiment in [5], the impact force was only applied on the capacitor tank, and the vibration of the capacitor elements under electromagnetic force and the vibration propagation inside capacitor were not taken into account.

In spite of the numerous studies on capacitor noise in HVDC systems, there still lack convincing and satisfying methods for noise level prediction. In this paper, a new noise level calculation approach based on electro-mechanical frequency response function (EMFRF), which is obtained from impulse current experiment, is presented and verified. The paper is organized as follows: In the first section (Noise Prediction Method Based on EMFRF), the definition of EMFRF is given and the noise-level prediction procedure is described. The second section discusses the obtaining of EMFRF from impulse current experiment. In the third section, capacitor noise level is predicted using the presented method and measured experimentally. The prediction results are compared with the experimental data so as to verify the effectiveness of the presented prediction method.

## Methods

### Generation of audible noise

**Figure 1:**
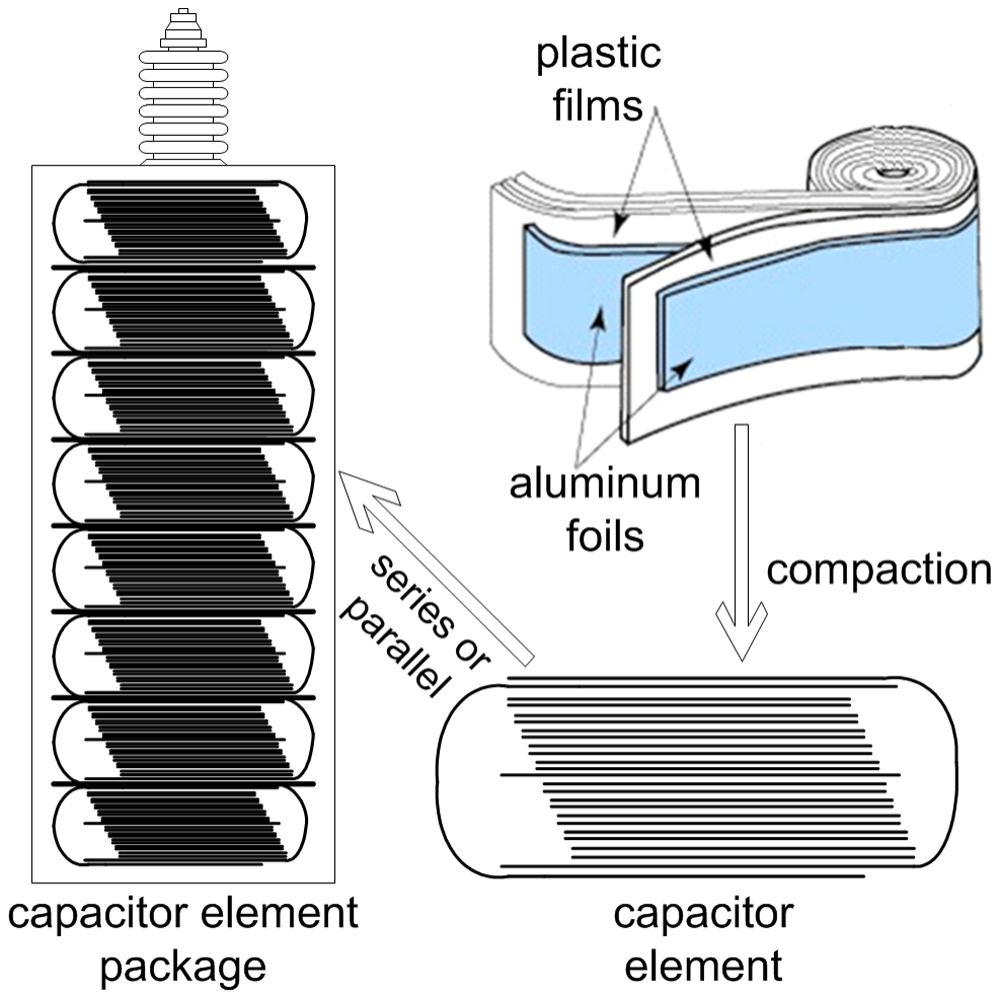
Structure of can-type capacitor.

In general, it is the can-type all-film capacitors that are used in HVDC systems. A capacitor unit is consisted of a steel-covered can and two bushings, the structure of which is shown in Figure 1. The can is filled with oil and contains a capacitor element package formed by a number of capacitor elements connected in series or parallel. The capacitor element is made by winding two aluminum foils and a number of plastic or paper films.

When voltage is applied on the capacitor, all aluminum foils are energized and nearly all plastic films are in force. Ac capacitor voltages will generate time-varying forces that lead to vibrations. The force in the capacitor element package finally causes vibrations of the steel enclosure of the capacitor unit and thus generates acoustic airborne sound [1], [6].

### Definition of EMFRF

A capacitor is assumed to be a linear mechanical system, which can be described by frequency response function 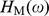 as follows:
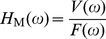
(1)where 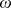 is the vibration angular frequency, 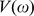 is the vibration velocity response of the capacitor tank in frequency domain, and 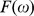 is the attractive electric force in frequency domain. As analyzed in [5], the attractive electric force 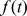, which is the motivation of capacitor vibration, is proportional to the square of the voltage applied on the capacitor. This relation is expressed as
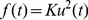
(2)where 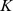 is the proportional coefficient, and 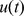 is the ac voltage applied on the capacitor. As a result, the system composed of the vibration response of the capacitor tank and the squared voltage applied on the capacitor is also linear. A frequency response function containing both electrical and mechanical characteristics is therefore employed to describe the system:
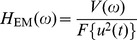
(3)where 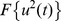 is the spectrum of the square of the voltage. The function 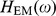 is named electro-mechanical frequency response function (EMFRF).

### Noise level prediction method based on EMFRF

Firstly, the voltage applied on a capacitor is calculated from the capacitance of the capacitor and the current flowing through it. The spectrum of the square of the voltage can be obtained by using Fourier transform method. This step has been described in [1].

Secondly, EMFRF of the capacitor, 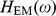, is obtained by impulse current experiment, which will be described in the section of “Obtaining of EMFRF from Impulse Current Experiment”.

Then the corresponding vibration velocity response of the capacitor tank is calculated by
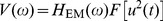
(4)


Finally, the audible noise level can be predicted from the vibration velocity. Suppose that the vibration velocity of a capacitor surface is v, and then its radiated sound power 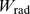 is
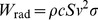
(5)where 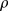 is the air density, c is the sound velocity in air, S is the area of the surface radiating sound, v is the vibration velocity, and 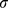 is the radiation ratio (no unit) [1]. The size of vibration source is much smaller than the main vibration wavelength. Therefore, according to [7], can be estimated by (6), which is demonstrated in Appendix S1.
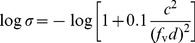
(6)where d is the feature size of sound source, 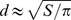, and 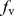 is the vibration frequency.

Meanwhile, the sound power level 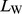 in the unit of decibels (dB) is defined by
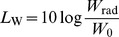
(7)where 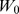 is the sound reference power of 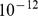 W [1].

Substitute (5) into (7), we have
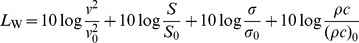
(8)where 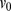 is the reference velocity of 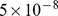 m/s, 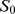 is the reference area of 1 m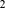, 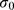 is the reference radiation ratio of 1, and 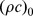 is the reference air sound impedance of 400 kg/(m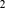 s).

In a semi-anechoic room (a room where sound reflections only come from the floor because the walls and ceiling are absorbent), the relation between sound power level 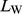 and sound pressure level 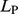 is
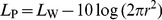
(9)where r is the distance from the measurement point to the sound source in the unit of m [1].

**Figure 2:**
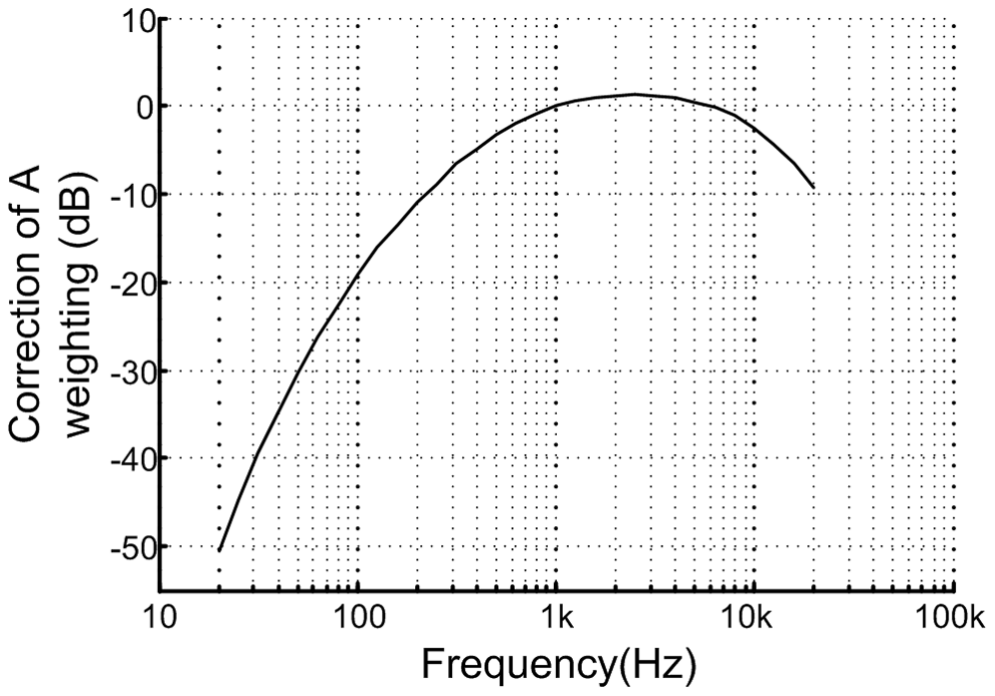
Frequency characteristics of A-weighting network.

Generally, A-weighted sound pressure level is widely used to evaluate the strength of noise. The frequency characteristics of A-weighting network are shown in Figure 2. Measurement adopting “A-weighting” is in the unit of dB(A), and generally agrees with people's assessment of “loudness”. The A-weighted sound pressure level 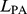 is calculated from the sound pressure level at each frequency by
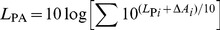
(10)where 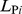 is the sound pressure level (in dB) at the ith frequency band, 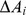 is the A-weighting gain (in dB) at the ith frequency band [1].

**Figure 3:**
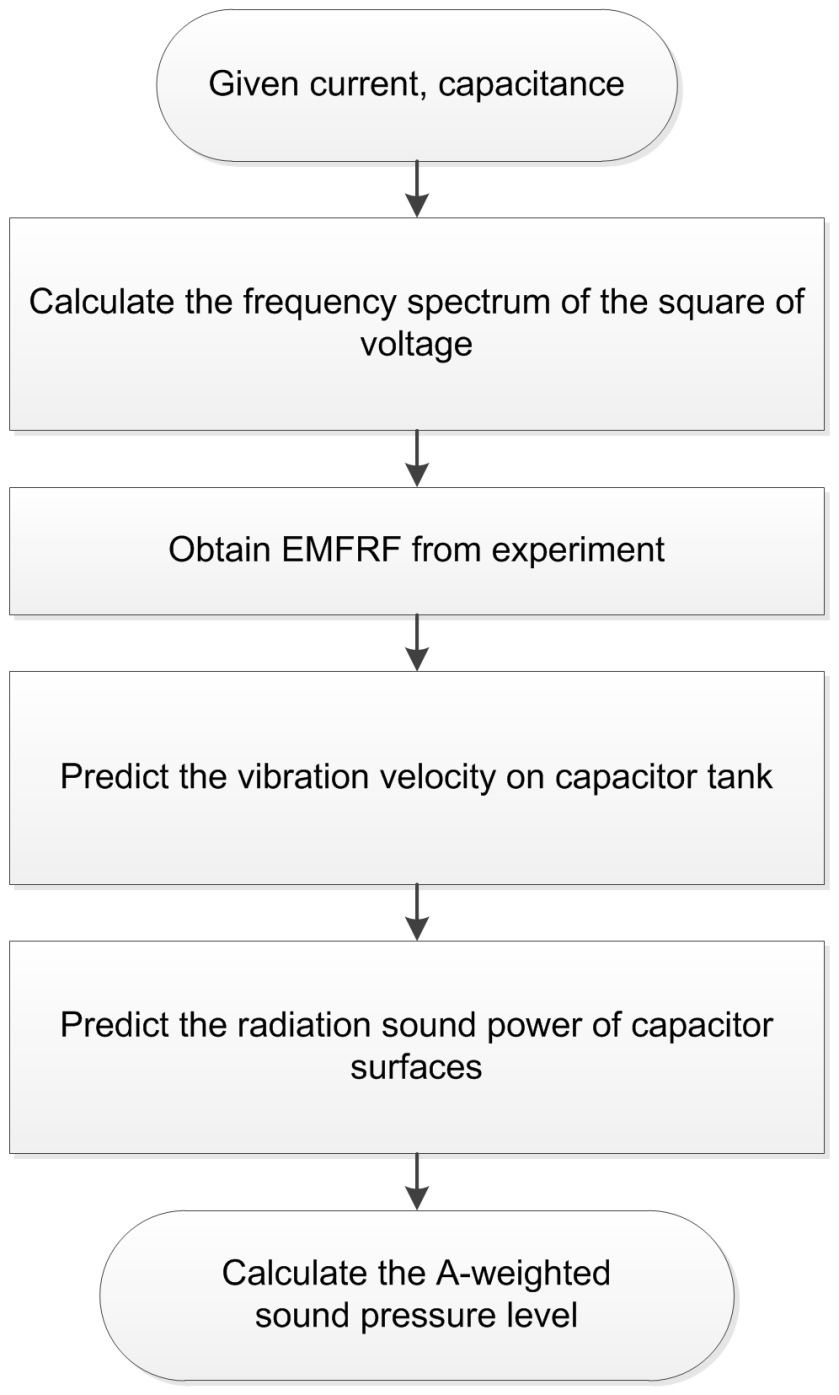
Flowchart of the noise level prediction based on EMFRF.

The flowchart of audible noise level prediction based on EMFRF is shown in Figure 3.

### Experimental system for measuring EMFRF

In order to learn the electro-mechanical characteristics of a capacitor in wide frequency band, impulse current experiment is employed to obtain EMFRF.

**Figure 4:**
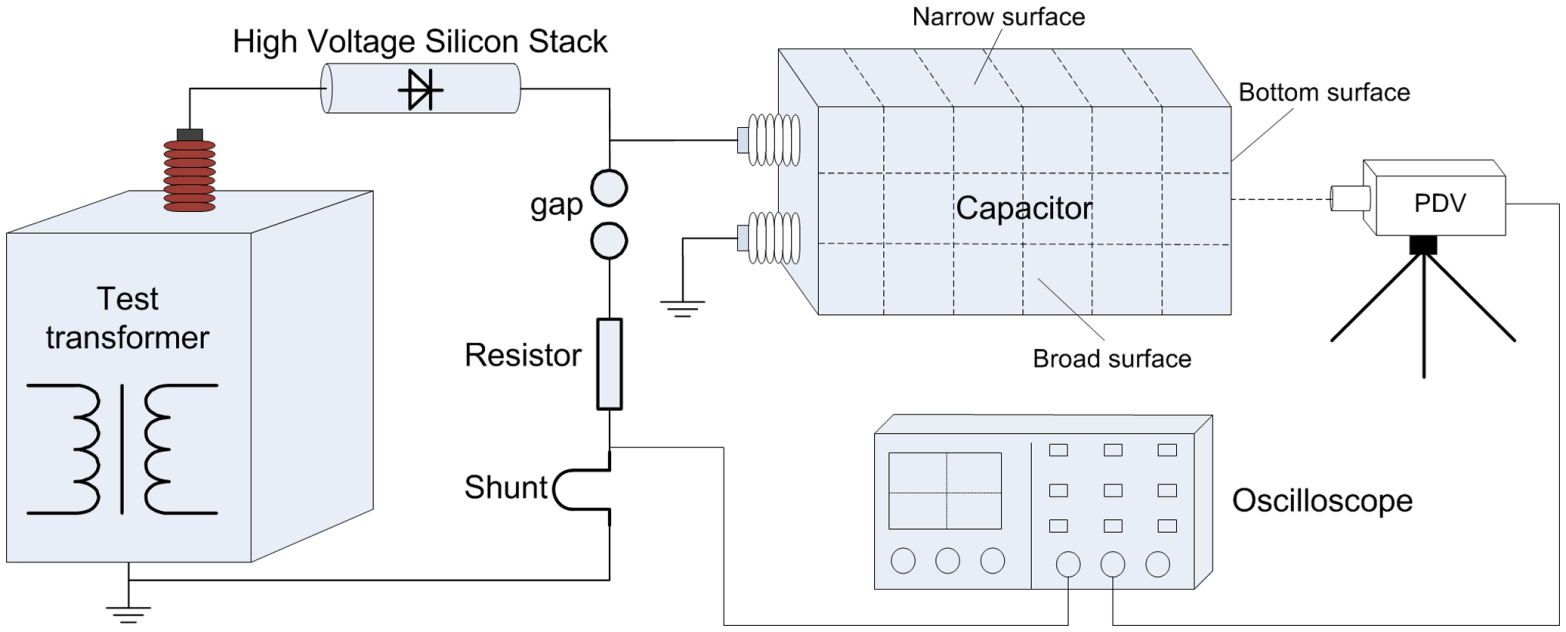
Experimental system for measuring EMFRF.

The experimental system for measuring EMFRF is shown in Figure 4. The experiment procedure is similar to that of the short-circuit discharge test in type tests of capacitors [8]. The capacitor is charged to a voltage 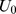 by the half-wave rectifier consisted of test transformer and high voltage silicon stack. The sphere gap is triggered and the capacitor discharges through a small resistor. Simultaneously, the vibration velocity of capacitor tank is measured by a portable digital vibrometer (PDV), and the impulse current applied to the capacitor is measured by the shunt. Both the impulse current signal and the vibration velocity signal are acquired by the oscilloscope.

The parameters of the devices are as follows:

- **Shunt:** Resistance of 0.00184 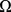,
- **PDV:** PDV-100 vibrometer manufactured by Polytec GmbH, working frequency range of 0-22 kHz, propagation delay of approximately 1 ms, measurement range of 20 mm/s, 100 mm/s or 500 mm/s (adjustable via display), low pass filter cutoff frequency (0.1 dB) of 1 kHz, 5 kHz or 22 kHz (adjustable via the display),
- **Oscilloscope:** DPO4054 oscilloscope manufactured by Tektronix, bandwidth of 500 MHz; sampling rate of 5 GS/s, record length of 20 M,
- **Capacitor:** Rated voltage of 12 kV, rated capacity of 417 kVar, measured capacitance of 9.46 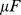.

**Figure 5:**
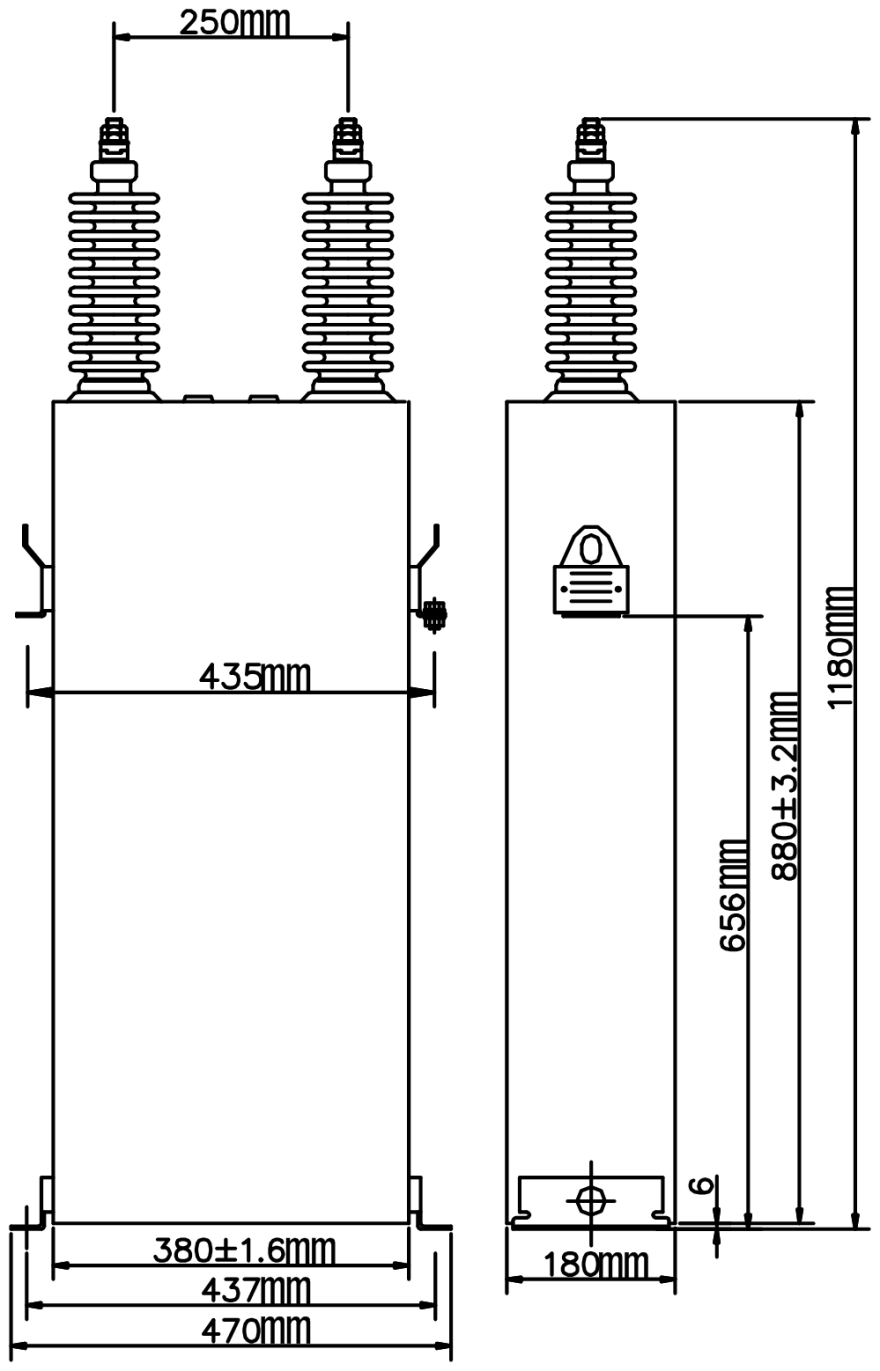
Configuration and dimension of the capacitor.

Configuration and dimension of the capacitor are shown in Figure 5. The surface where the bushings are installed is appointed as top surface, and the opposite is the bottom surface. The other four surfaces are called narrow surface or broad surface according to their breadth.

**Figure 6:**
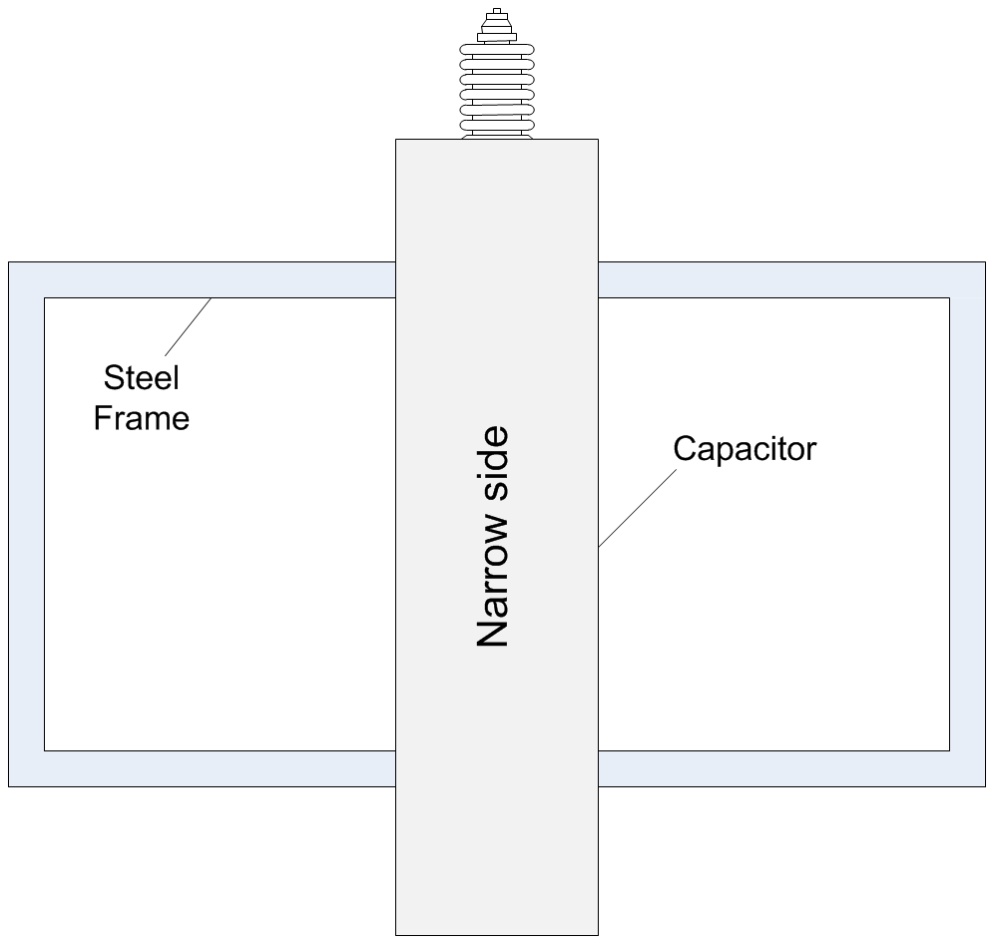
Fixing of the capacitor (top view).

The capacitor is fixed on a steel frame with the narrow side upward as in the field (Figure 6).

### Calculation of the spectrum of the squared voltage

**Figure 7:**
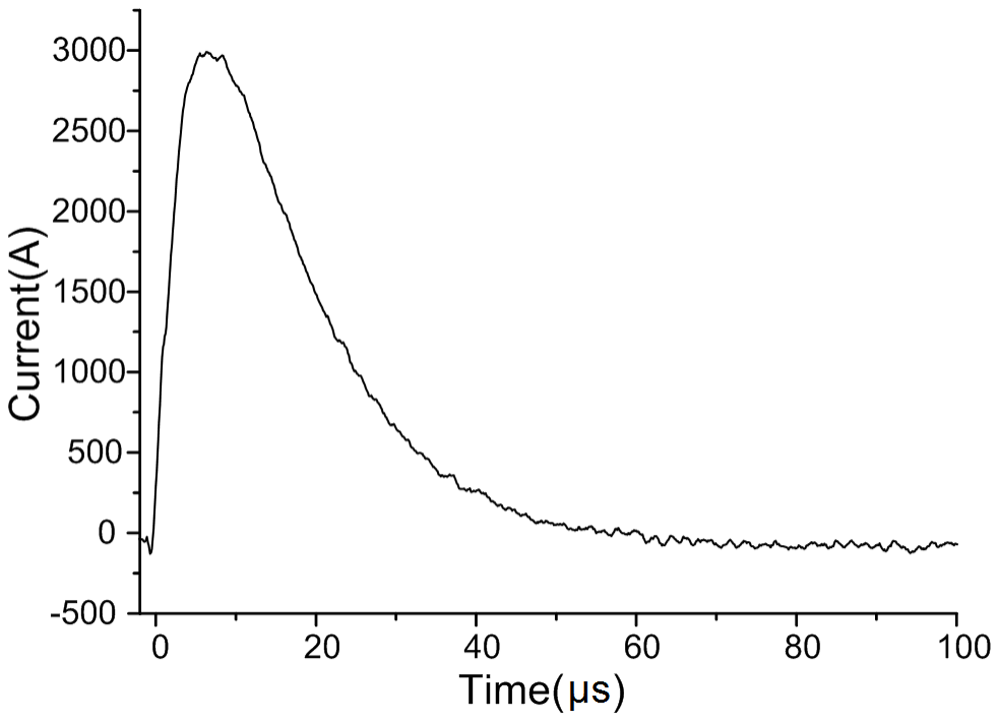
Waveform of the impulse current.

The small resistor is adjusted in advance to make sure that the impulse current contains abundant frequency components. The waveform of the impulse current is shown in Figure 7. The voltage on the capacitor is the integral of the current, which is
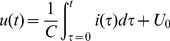
(11)


**Figure 8:**
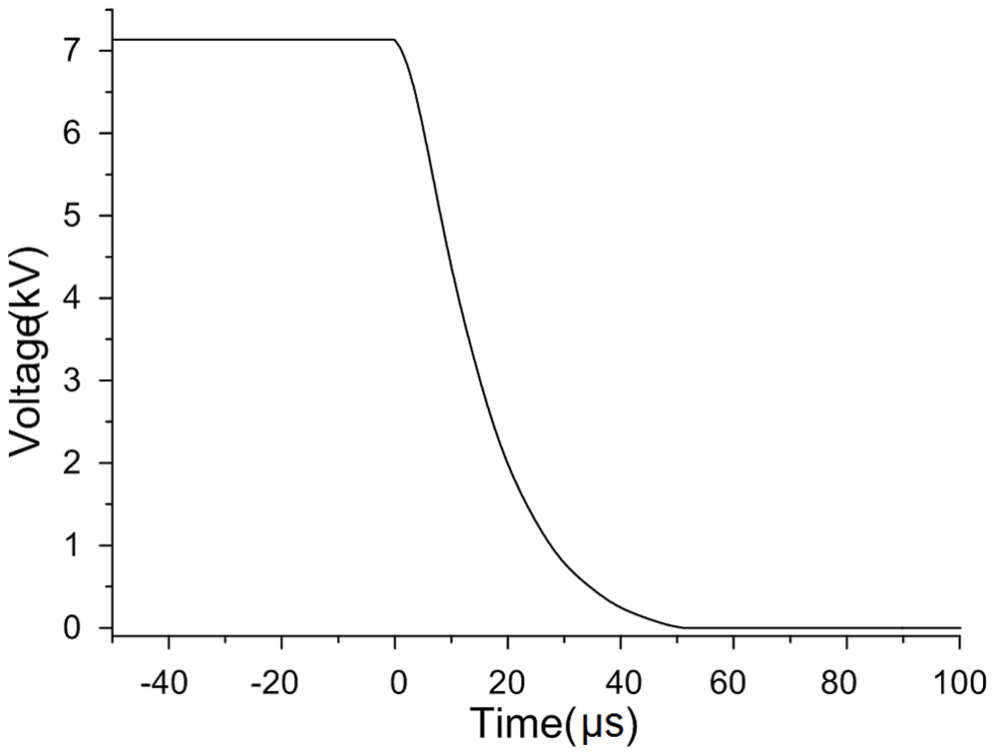
Waveform of capacitor voltage.

As shown in Figure 8, the waveform of the voltage has a steep trailing edge when the impulse current flows through the capacitor. Obviously, the square of the voltage is infinite and aperiodic in time domain, so its spectrum cannot be calculated directly by Fourier transform. However, the derivative of the squared voltage is finite and the spectrum can be calculated. The spectrum of the squared voltage can be then obtained based on the derivative property of Fourier transform.

The derivative of the square of voltage is
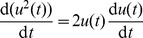
(12)


The capacitor current is the derivative of the voltage, which is
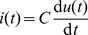
(13)


Synthesize (12) and (13), we can get
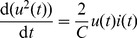
(14)


According to the derivative property of Fourier transform, if 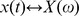, there is 
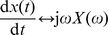
. As a result, the Fourier transform of 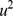 can be calculated as
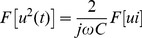
(15)


### Calculation and analysis of EMFRF

**Figure 9:**
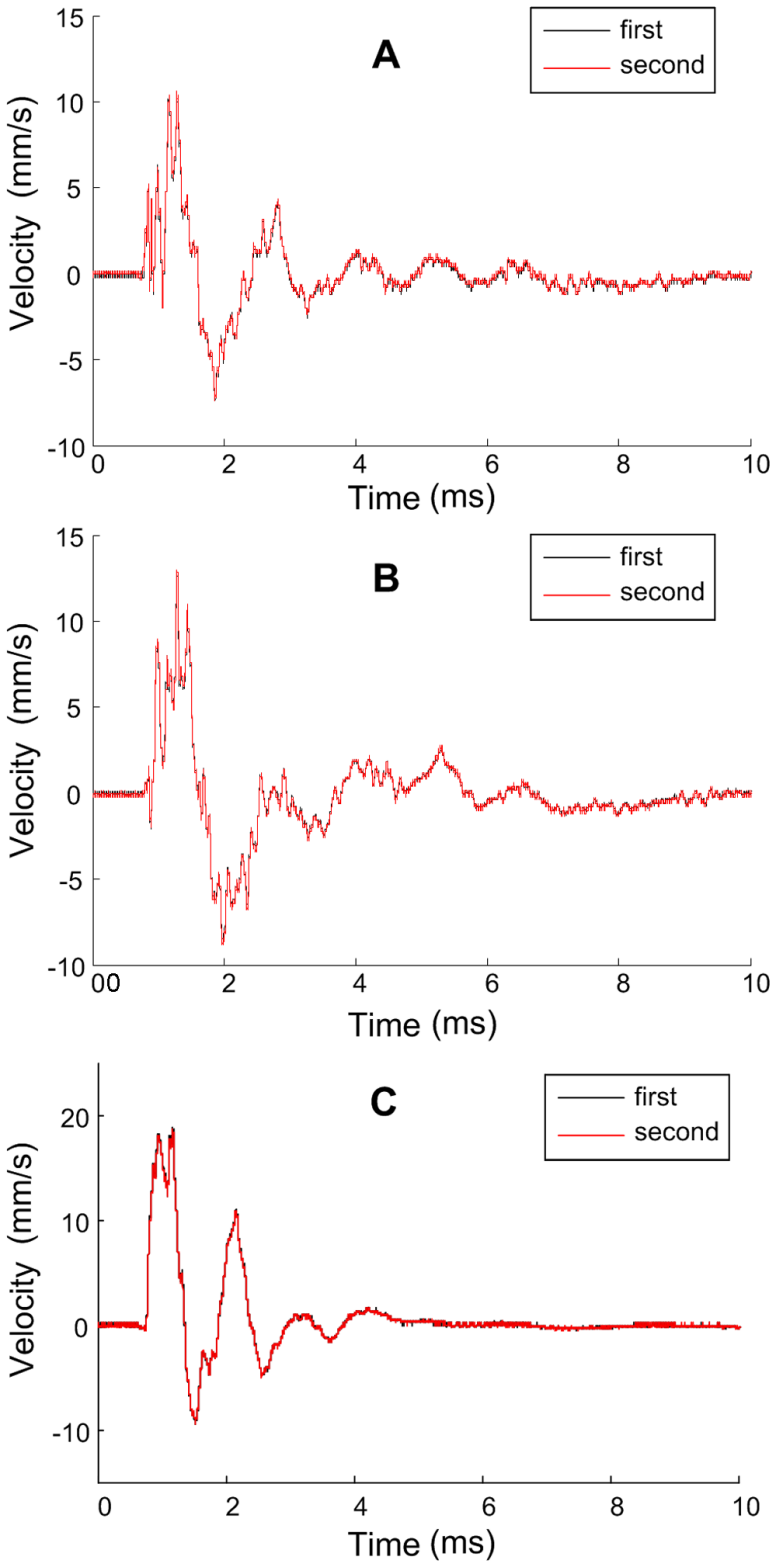
Two vibrations measured at different time. A Broad surface, B Narrow surface, and C Bottom surface.

A number of n equally spaced points are marked over the surfaces of the capacitor tank. The impulse current is applied to the capacitor repeatedly. Simultaneously, the vibration velocities of the marked points are recorded sequentially. The vibrations at each point are measured at least two times to avoid the influence of random error on the measurement. In fact, at all the measurement points, the two vibrations measured at different time are exactly the same as shown in Figure9, which means capacitor responses identically under same electric excitations and the reproducibility of the calculation method is satisfying.

Suppose that when the impulse current 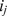 is applied, the recorded vibration velocity at the 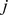
th point is 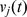 and the voltage is 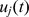. Synthesize (3) and (15), we have
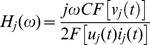
(16)where j = 1,2,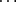,n.

**Figure 10:**
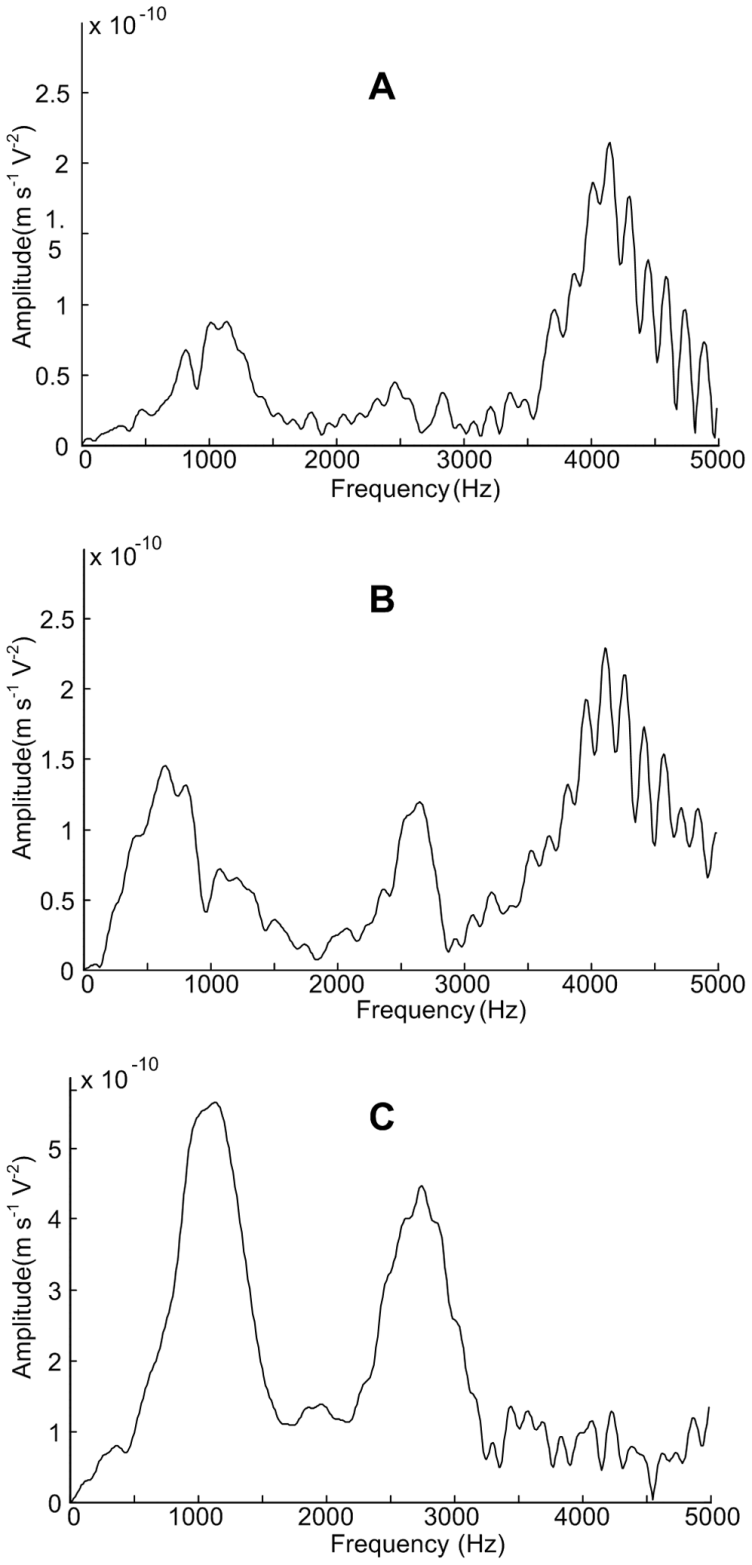
EMFRF of capacitor surfaces. A Broad surface, B Narrow surface, and C Bottom surface.

The magnitude plots of EMFRF for the broad, narrow and bottom surfaces of the tested capacitor are shown in Figure 10. The plots indicate that the vibration responses of side surfaces are quite similar, and that of bottom surface is much different. The magnitude of the side surface EMFRF is very small for low frequency (under 3 kHz). When the frequency is above 3 kHz, there are a large number of resonant frequencies, represented by the peaks of EMFRF magnitude. However, the magnitude of EMFRF of bottom surface is big under 3 kHz and small above 3 kHz. The peak value of bottom surface is about 4-6 times that of side surfaces, which is consistent with the conclusion that the bottom surface is the main source of noise radiation [1].

In this experiment, the impulse current flows through the capacitor and the capacitor elements are motivated to vibrate, so that the obtained EMFRF contains the information of vibration generation and propagation in addition to the mechanical features of the capacitor. Compared with the calculation method presented in [5], the method proposed here has a better accuracy. In [5], frequency response functions which describe the natural vibration characteristics of capacitor tank were obtained from impact hammer experiment. The impact force was only applied on the surface of capacitor tank in that experiment, so the vibration of the capacitor elements under electromagnetic force and the vibration propagation inside capacitor were not taken into account.

### Laboratory measurement of capacitor noise

The noise levels of the capacitor under certain operating conditions are measured in laboratory. The experimental data are compared with the calculation results of the presented prediction method to verify the effectiveness of the method.

**Figure 11:**
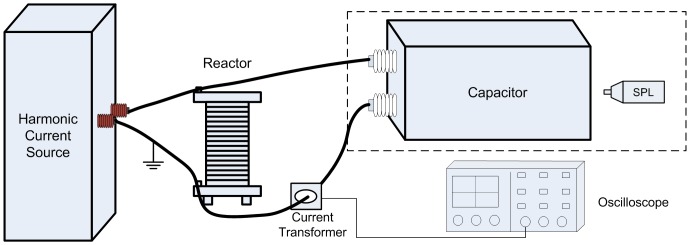
Noise measuring system.

The noise measuring system, shown in Figure 11, consists of current measuring part and noise measuring part. The main circuit is made up of the reactor, the capacitor and the harmonic current source. The reactor and the capacitor form the current resonance circuit. The frequency of the harmonic current source can be controlled within the range of 0-600 Hz. A sound pressure spectrum analyzer, marked as SPL in Figure 11, is used to record the audible noise data. The capacitor and the analyzer are placed in a semi-anechoic room to avoid interference from environmental noise.

The parameters of the devices are as follows:

- **Sound pressure spectrum analyzer:** NA-28 sound-level meter and the 1/3 octave band real-time analyzer manufactured by RION in Japan, sensitivity of -27 dB and measurement range from -25 dB to 130 dB,
- **Semi-anechoic room:** Space size of 6.3 m5.5 m5.4 m, frequency range from 100 Hz to 10 kHz for free field, background noise of 23.9 dB.
- **Oscilloscope:** DPO4054B oscilloscope manufactured by Tektronix, bandwidth of 500 MHz, sampling rate of 5 GS/s, record length of 20 M.

The sound pressure level of the filter capacitor is measured under five different currents. The frequencies and amplitudes of the test currents are shown in Table 1. To avoid the impact of the mechanical fixing on the radiating noise level, the capacitor is fixed in the same way as in the experiment for measuring EMFRF.

**Table 1:** Test current.

Frequency/Hz	150	250	350	450	550
Current/ 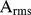	34.75	34.75	34.75	34.75	34.75

**Figure 12:**
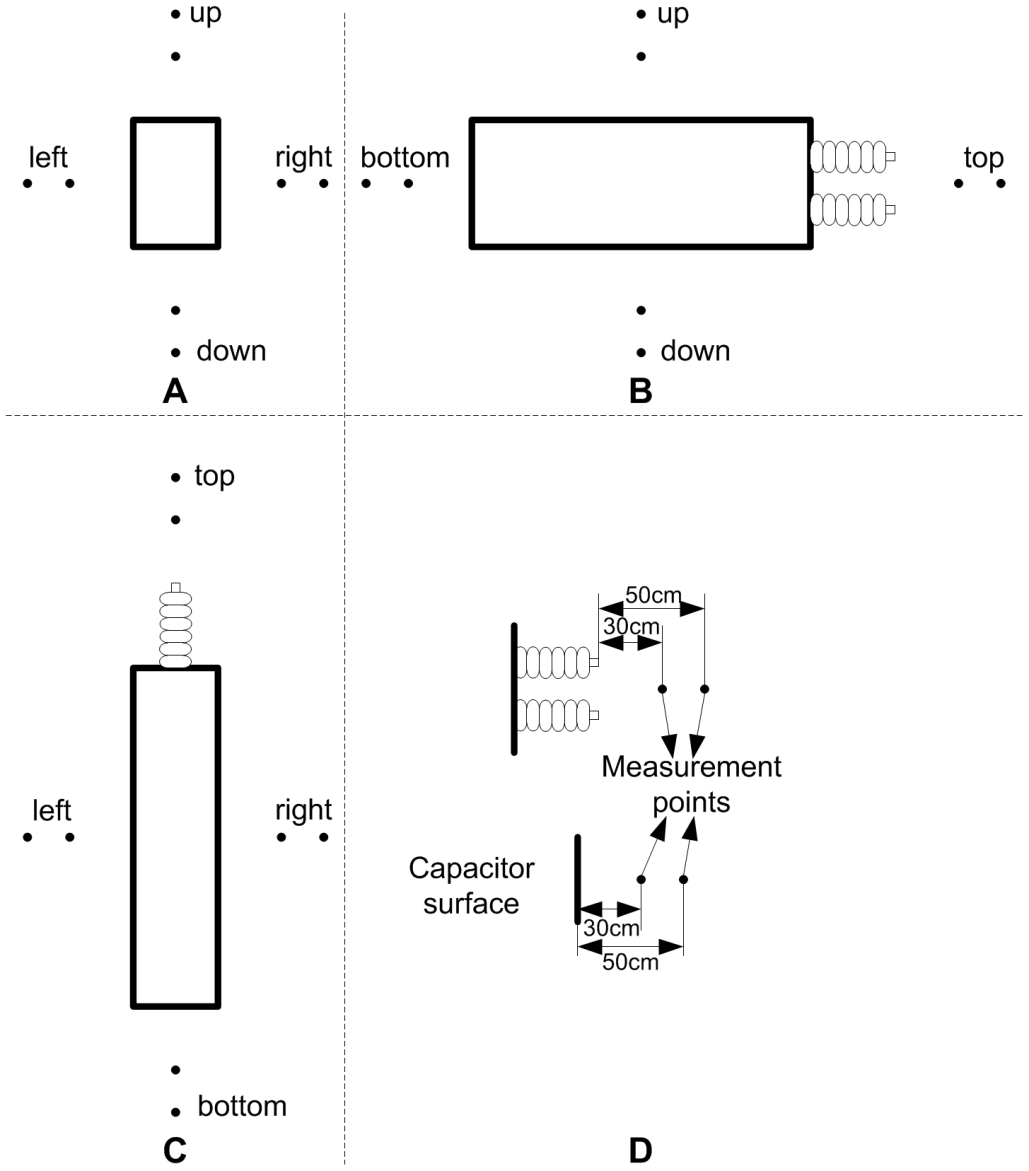
Measurement points for audible noise. A Front view, B Right View, C Top view, and D measurement-point arrangement.

Measurement points for the audible noise are arranged at 30 cm and 50 cm away from the center of each surface, as shown in Figure 12. Compared with the arrangement in [9], the arrangement in this paper adds the measurement points at 30 cm to verify the effectiveness of the presented prediction method under different distances. The data of current flowing through the capacitor is acquired simultaneously with audible noise at the measurement points.

### Calculation of sound pressure level via the presented prediction method

At a given current angular frequency 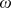 and current amplitude 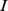, the voltage of the capacitor is a single frequency signal with the angular frequency of 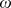. Its amplitude can be calculated by
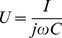
(17)


The square of the voltage can be expressed as
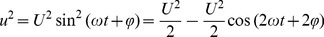
(18)


It can be seen that the square of the voltage contains a dc component and a sinusoidal component with the angular frequency of 2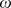 and the amplitude of 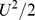.

As expressed in (4), the vibration responses of each surface equal the frequency domain product of the squared voltage and the calculated EMFRF. The vibration velocity at each of the marked points for calculating the noise level can be predicted. The geometric average of the predicted velocities at all the points over each surface is adopted as the vibration velocity of the corresponding surface:
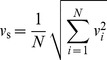
(19)where 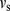 is the surface vibration velocity, N is the number of measurement points, 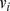 is the predicted velocity at the 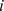
th point. The calculation results are shown in Table 2.

**Table 2:** Calculated vibration velocity.

			Vibration velocity/mm/s		
Current frequency/Hz	Voltage/kV	Vibration frequency/Hz			
			
			bottom surface	broad surface	narrow surface
150	3.9	300	0.59	0.15	0.17
250	2.3	500	0.29	0.11	0.12
350	1.7	700	0.21	0.12	0.08
450	1.3	900	0.32	0.08	0.03
550	1.1	1100	0.25	0.04	0.02

Based on the calculated vibration response, the sound pressure level of each surface can be predicted by (8) to (10).

## Results and Discussion

### Comparison of Measurement and Prediction

The measurement results and prediction results are compared, as shown in Table 3 and Table 4. The predicted noise level is close to the measured data at most points, suggesting that the presented method can effectively predict the noise level of capacitors in HVDC systems. There exist certain differences at a couple of points under certain excitations, for example, the 30 cm point of bottom surface under 350 Hz excitation and the 50 cm point of narrow surface points under 450 Hz excitation. The discrepancies are mainly caused by the simplifications in the model. The geometric average velocity of all the points on each surface is taken as the vibration velocity of the whole surface, which may cause errors in calculation. In addition, it is assumed that the capacitor in our study is a linear mechanical system, but it is not perfectly linear actually. What's more, equation (9) is valid for far field conditions, but the measurements and the calculations in this study are conducted for the near field. The hemispherical sound propagation mode is chosen to simplify the model [1]. Comparison of the calculation results and the measurement results indicates that the simplification is acceptable, but it still may bring errors into calculation. Lastly, the capacitor current in the noise measuring experiment may also vary with the power grid fluctuation, making the measurement results deviate from the real values.

**Table 3:** Comparison between the Measured and the Predicted Noise Levels at 30(dB(A)).

Frequency of current/Hz	Surface	Measurement	Prediction	Deviation
	Bottom	56.4	56.9	0.9%
150	Broad	50.8	53.3	4.9%
	Narrow	51.6	51.1	1.0%
	Bottom	61.4	59.7	2.8%
250	Broad	56.2	57.4	2.1%
	Narrow	56.0	56.0	0.0%
	Bottom	67.3	63.6	5.5%
350	Broad	59.2	60.4	2.0%
	Narrow	58.1	55.7	4.1%
	Bottom	69.9	68.4	2.1%
450	Broad	63.0	61.1	3.0%
	Narrow	54.8	53.2	2.9%
	Bottom	65.1	65.3	0.3%
550	Broad	56.8	55.9	1.6%
	Narrow	46.8	46.0	1.7%

**Table 4:** Comparison between the Measured and the Predicted Noise Levels at 50(dB(A)).

Frequency of current/Hz	Surface	Measurement	Prediction	Deviation
	Bottom	52.5	52.4	0.2%
150	Broad	47.2	49.4	4.7%
	Narrow	46.6	48.3	3.6%
	Bottom	56.1	56.3	0.4%
250	Broad	50.9	53.4	4.9%
	Narrow	54.0	52.50	2.8%
	Bottom	61.9	60.2	2.7%
350	Broad	55.4	56.5	3.8%
	Narrow	55.2	53.7	2.7%
	Bottom	64.0	63.0	1.6%
450	Broad	59.2	56.9	3.9%
	Narrow	52.7	49.2	6.6%
	Bottom	62.1	61.9	0.3%
550	Broad	56.3	54.6	3.0%
	Narrow	45.6	44.1	3.3%

### Limitations of the Study, Open Questions and Future Work

In this paper, the deviations in the comparison are just qualitatively analyzed. Some possible factors causing the discrepancies are given. In the future, we plan to quantify the errors caused by these factors and amend the calculation method accordingly. The nonlinearity of the system will be modeled and calculated to evaluate the nonlinear error in the calculation. The error brought by the substitution of the geometric average velocity for the actually velocity will be analyzed by the simulation software Sysnoise. In Sysnoise, the two modes for velocity boundary should be both employed, and the discrepancy in the calculation results will show the quantitative error. At last, the noise will be measured continuously in the future research and stable results will be chosen to lower the measurement error.

The predicted noise should be compared with the measured result at a distance much further than 50 cm since the noise level in the nearby residential district is the greatest concern. However; the noise measurement distance is restricted by the size of the semi-anechoic room in this paper. According to ISO 3745-2003 [10], the noise measurement is conducted in a semi-anechoic room to provide a free field over a reflecting plane without environment interference. The size of the semi-anechoic room is 6.3 m5.5 m5.4 m. As a result, the measurement distance cannot be any further. Nevertheless, we believe that the comparisons at the two distances are convincing enough to prove the effectiveness of the proposed method. In the further research, we plan to compare the predicted noise with the sound field simulation result at a further distance to refine our work.

Only the noise prediction method for single capacitors is proposed and verified in this paper, the calculation method for multiple capacitors is not involved. To do this, it should be firstly proved that the capacitors of a same type radiate same noise under same excitations. In [5], the uniformity has been assumed because the inside structure is uniform, which is theoretically reasonable. Besides, the discrepancy between the noises radiated from capacitors of a same type can also be investigated by experiment to prove the uniformity, which will be our future work. Another trouble in the calculation of multiple-capacitors noise is the superimposition of the noise radiated from independent sources. The sound field becomes complicate in the case of multiple sources, so it still needs further study.

## Conclusions

In this paper, a novel noise level prediction method is presented, which is based on a frequency response function considering both electrical and mechanical characteristics of capacitors. The “electro-mechanical frequency response function (EMFRF)” is defined as the frequency domain quotient of the vibration response and the squared voltage. EMFRF can be obtained from impulse current experiment. The vibrations of the capacitor surfaces under given excitations can be predicted based on the measured EMFRF, and the noise levels can then be calculated. The prediction results are compared with the measured data in a laboratory experiment. In spite of some differences, the predicted noise levels are close to the measured values at most measurement points. This indicates that the noise level prediction method for capacitors in HVDC converter stations presented in this paper is effective.

The impulse current experiment in this study uses electric excitations, which is the same as the practical situation. Therefore, the EMFRF obtained from the experiment can reflect not only the natural mechanical characteristics but also the electrical characteristics of capacitors. EMFRF builds the connection between electrical excitations and vibration responses of capacitor tanks, which is not achieved in other methods. This is a great progress in noise level prediction for capacitors in HVDC converter stations.

## Supporting Information

Appendix S1 demonstration of calculation formula for radiation ratio
(DOC)
