# Supplementary material for: A Noise Level Prediction Method Based on Electro-Mechanical Frequency Response Function for Capacitors
Source: PLoS One. 2013 Dec 9;8(12):e81651. doi: 10.1371/journal.pone.0081651 (PMC3857221; doi:10.1371/journal.pone.0081651)
Supplement: Appendix S1 — demonstration of calculation formula for radiation ratio (DOC) [file pone.0081651.s001.doc]

# **Appendix S1：Demonstration of Calculation Formula for Radiation Ratio**

If the size of vibration source is much smaller than the main vibration wavelength, the vibration source can be regarded as spherical source. For a spherical sound source,

$$\sigma=\frac{k^{2}a^{2}}{1+k^{2}a^{2}}$$

where *k* is the wavenumber, $k=2\pi f_{v}/c$; *a* is the radius of the spherical source, $a=d/2$. Substitute *k* and *a* into , we get

$$\sigma=\left[1+\frac{c^{2}}{\pi^{2}(f_{v}d)^{2}}\right]^{-1}$$

Therefore

$$\log\sigma\approx-\log\left[1+0.1\frac{c^{2}}{(f_{v}d)^{2}}\right]$$
